# Pre-treatment with chemotherapy can enhance the antigenicity and immunogenicity of tumours by promoting adaptive immune responses

**DOI:** 10.1038/sj.bjc.6605465

**Published:** 2009-12-08

**Authors:** W M Liu, D W Fowler, P Smith, A G Dalgleish

**Affiliations:** 1 Section of Oncology, Division of Cellular and Molecular Medicine, St George's University of London, London, UK

**Keywords:** immunotherapy, HLA1, chemotherapy, dendritic cells, immunovisibility

## Abstract

**Background:**

Some cancer patients are immuno-compromised, and it has been long felt that immune-intervention is not compatible with standard chemotherapies. However, increasing evidence suggests that standard chemotherapy drugs may stimulate beneficial changes in both the immune system and tumour.

**Methods:**

We have assessed the expression of human leucocyte antigen class 1 (HLA1) on tumour cells before and after chemotherapy agents (cyclophosphamide, oxaliplatin or gemcitabine). In addition, we show that chemotherapy-stressed tumour cells may release cytokines that enhance the interactions between dendritic cells (DCs) and T cells into growth media.

**Results:**

Here we report that some chemotherapy agents can increase HLA1 expression in tumour cells, even when expression is low. Increases were associated with killing by cytotoxic T cells, which were negated by HLA1-blockade. Furthermore, T-cell function, as indicated by increased proliferation, was enhanced as supernatants derived from tumours treated with chemotherapy augmented DC-maturation and function.

**Conclusion:**

There is evidence that a facet of immune surveillance can be restored by appropriate chemotherapy agents. Also, tumours exposed to some chemotherapy may secrete cytokines that can mature DCs, which ultimately enhances T-cell responses.

Cancer is associated with chronic inflammation and marked systemic immune suppression (O'Byrne and Dalgleish, 2001; Dalgleish and O'Byrne, 2002, 2006; Evans *et al*, 2006). Indeed, the evasion of immune surveillance and negation of its function are hallmarks or cancer. These aspects are widely overlooked (Hanahan and Weinberg, 2000), and the development of new therapies in cancer have primarily focussed on tumour killing and disturbing tumour-microenvironment interactions. These typical therapeutic approaches have resulted in impressive activities, and as our understanding of the pathogenetic pathways underlying cancer improve, new drugs that correct specific molecular defects in cancer will be formulated to fuel further successes (Workman and Kaye, 2002). Drawbacks exist, however, that limits the long-term efficacy of these drugs, such as the gradual loss of activity (Liu, 2008). These failings stem from the direct effect of the drugs, and develop gradually, as the cancer adapts to treatment and develops drug resistance. One way to prevent these adverse events is to ensure that tumour cells are cleared more efficiently and residual tumour cells are minimised. For this reason, the restoration of a competent immune system that is innately programmed to remove foreign material is an attractive aim (Bhardwaj, 2007; Liu *et al*, 2009).

Cytotoxic chemotherapeutic drugs affect rapidly growing cells, and as a consequence cause collateral damage to cells of the immune system. In this regard they are considered immunosuppressive. However, there is increasing evidence to suggest that some cancer chemotherapies may actually aid immunotherapy by activating the immune system rather than suppressing it (Chaudhuri *et al*, 2009). This is supplementary to the conventional cytotoxic effects, and may be a product of enhancements in the adaptive response. For example, the nucleoside analogue gemcitabine (GEM), in addition to its apoptotic effects, selectively promotes the cell-mediated immune response over the humoral immune response by selectively inhibiting B-cell proliferation (Nowak *et al*, 2002), decreasing memory T cells, and promoting the activation of naive T cells (Plate *et al*, 2005) and function of CD8+ T cells (Suzuki *et al*, 2005). Immunopotentiation is also achieved in part by the inhibitory effect of GEM on myeloid-derived suppressor cells (Bronte *et al*, 2000). Furthermore, maximising the interactions between T cells and professional antigen presenting cells (APCs), such as dendritic cells (DCs), have been shown to enhance adaptive responses, which ultimately leads to the elimination of tumour (Steinman and Banchereau, 2007). The maturation of DCs has a key role in initiating T-cell responses as they possess the ability to initiate primary adaptive immune response through the capture, processing and presentation of antigen to naive CD4+ and CD8+ T cells. These have pivotal roles in the induction of T-cell-mediated anti-tumour responses *in vivo*.

In addition to the effects on immune cells, the process of immunosurveillance can also be a target for chemotherapy; indeed, the ability to evade immunity is a regular feature of cancer cells. The molecular transformations that occur during oncogenesis, which cause cancer cells to be pro-survival and anti-apoptotic are the same ones that interfere with immune responses against tumour cells (Fuchs and Matzinger, 1996; Pardoll, 2003). For instance, dysregulation of the RAS and mitogen activated protein kinase signalling pathways that promote anti-apoptotic and pro-survival behaviour in some cancers (Dhillon *et al*, 2007; Roberts and Der, 2007) can also interfere with human leucocyte antigen class 1 (HLA1) antigen processing on tumour cells. As cytotoxic T cells are restricted by HLA1 and kill tumour cells only in the presence of HLA1, tumours lacking this antigen may become undetectable to immune cells. It is predicted that restoration of HLA1 expression could re-initialise immune visibility in tumour cells.

As part of our ongoing studies to investigate the effect of chemotherapy on immune function, we explored the effect of supernatant derived from tumour cells exposed to chemotherapy, on DC function, to test the hypothesis that chemotherapy-stressed tumour cells secrete cytokines and other factors that promote the antigen presenting behaviour of DCs. In addition, we explored the role of chemotherapy on the HLA1 expression on tumour cells to see if restoration of HLA1 expression on tumour cells may re-engage immune-cell function and enhance tumour cell death.

## Materials and methods

### Reagents

Cyclophosphamide (CPM; Sigma, Dorset, UK), GEM (Eli Lilly Pharmacy, St George's Hospital, London, UK) and oxaliplatin (OXP; Sigma) were dissolved in dimethyl sulphoxide (DMSO) to create 10 mM stock solutions that were maintained at −20°C for no longer than 4 weeks. For *in vivo* studies, the drugs were dissolved in 0.5% DMSO in phosphate-buffered saline (PBS) and stored at 4°C for the duration of the experiment. All controls used in our studies involved treatment with equal amounts of DMSO, the final concentrations of which were <0.1%.

### Cell culture

The human cancer cell lines A549 (lung), Caki2 (kidney), HCT116 (colon), MCF7 (breast) and PC3 (prostate) were obtained from the Cancer Research UK Cell Production Laboratories and maintained in culture medium supplemented with 10% (v/v) foetal bovine serum (FBS), 2 mM L-glutamine and 1 × penicillin/streptomycin (basal culture medium). All cell lines were incubated in a humidified atmosphere with 5% CO_2_ in air at 37°C, and discarded when the passage number exceeded 15.

To study the effect of CPM, GEM and OXP on cell growth, cells growing exponentially were added to 96-well plates at a density of 5 × 10^4^ per well. Drugs were then added to the wells, ensuring an equal volume of 200 *μ*l across the plate. Cell number was assessed at 72 h using the methylthiazoletetrazolium (MTT) assay, and the concentration of each drug required to reduce cell viability by 50% (IC50) was determined using the sigmoid Emax model as described previously (Liu *et al*, 2008).

### HLA1

Exponentially growing cells were reset in fresh culture medium at 2 × 10^5^ cells ml^−1^. Following a settling-in period of 24 h, cells were treated for 3 days with equi-active concentrations of CPM (10 *μ*M), GEM (1 *μ*M) or OXP (5 *μ*M). Exhausted culture medium (supernatant) was gently aspirated and used in our DC studies. Cells were harvested (1 × 10^5^) and washed twice in wash buffer (PBS containing 1% (v/v) FBS and 0.09% (v/v) NaN_3_), and then incubated with a fluorescein isothiocyanate-conjugated anti-HLA1 antibody (anti-HLA–ABC – 1 : 1000: BD Biosciences, Oxford, UK) for 30 min at 4°C. Acquisition of data was carried out within 1 h using a FACSCalibur (BD Biosciences). In all, 10 000 cells were analysed for each sample, and then the mean fluorescence intensity (MFI) of HLA1 was determined using the program WinMDI v2.9 (http://facs.scripps.edu/software.html).

### Cytotoxicity assay

T cells were isolated from pathologically healthy donor buffy coats (National Blood Service, London, UK) using positive cell isolation with magnetic beads coated with anti-CD3 (Miltenyi Biotec, Surrey, UK) according to manufacturer's instructions. CD3 purities, as assessed by flow cytometry, that were greater than 90% were used. Tumour cells pre-treated with CPM, GEM or OXP for 3 days were reset in culture medium at 1 × 10^4^ cells per well in a 96-well plate in the presence or absence of a HLA1 blocking antibody (1–10 *μ*g ml^−1^; Cambridge BioScience, Cambridge, UK). Cells were allowed to adhere before adding T cells at an effector : target ratio of 20 : 1. After a 24-h incubation period, cell-free media were removed for the assessment of lactate dehydrogenase (LDH) release using a proprietary assay kit (Cambridge BioScience). Non-adherent T cells were removed by washing twice in PBS and the tumour cell numbers were assessed *in situ* by the MTT assay.

### *In vivo* model

Female nude mice were purchased from and maintained by the Biological Research Facility in a pathogen-free environment at SGUL. Animals were acclimatised for at least 7 days before each experiment, and were used at the age of 9–13 weeks. All procedures were conducted in accordance with, and approved, by the Home Office of the United Kingdom.

Exponentially growing HCT116 cells were harvested, washed and re-suspended in PBS at a concentration of 1 × 10^7^ ml^−1^. Only cells with a viability of >90%, as assessed by trypan blue dye exclusion analysis, were used. Tumour cell suspension (300 *μ*l) was then injected subcutaneously into the dorsolateral flanks of the mice and allowed to establish. Upon reaching an approximate size of 5 mm × 5 mm, CPM (100 mg kg^−1^), GEM (50 mg kg^−1^) or OXP (10 mg kg^−1^) was administered daily by intra-peritoneal injection. Drugs were used at clinical achievable doses. Mice were killed 3 days post treatment, and tumour masses were resected and cells disaggregated before assessing HLA1 expression as described previously.

### Generating immature DCs

Peripheral blood mononuclear cells were isolated from pathologically healthy donor whole blood (National Blood Service) using Histopaque-1077 (Sigma). The mononuclear fraction was harvested and red blood cell contamination removed by incubation in hypotonic ammonium chloride. Cells were washed in PBS and platelet contamination removed by centrifugation at 200 **g** for 10 min, re-suspended at a concentration of 3 × 10^6^ ml^−1^ in basal RPMI-1640 culture medium and incubated for 2 h in a humidified atmosphere with 5% CO_2_ in air at 37°C to allow monocyte adhesion. After this time, the medium that contained non-adherent cells was aspirated from the culture flasks and stored at −80°C for the T-cell proliferation work. Fresh DC-maturing medium (culture medium containing 50 ng ml^−1^ interleukin-4 (IL4; Peprotech, London, UK) and 100 ng ml^−1^ granulocyte macrophage-colony stimulating factor (GMCSF; SGUL Pharmacy) was then added to the flasks containing the adherent monocytes. Flasks were returned to the incubator for a further 7 days and fed q.o.d. with DC-maturing medium. After this time, non- and loosely-adherent cells (DC fraction) were harvested and the purity assessed by CD11c/HLA–DR/CD14 immuno-discrimination by the flow cytometer. All fluorophore-conjugated antibodies were purchased from BD Biosciences.

### Stimulating DCs with tumour-derived supernatant

Immature DCs were reset at 1 × 10^5^ cells ml^−1^ in supernatants derived from A549 or MCF7 tumour cells cultured with CPM, OXP or GEM, and maintained in a humidified atmosphere with 5% CO_2_ in air at 37°C for 24 h. DCs were harvested, washed in wash buffer and incubated with a combination of phycoerythrin anti-CD80, allophycocyanin anti-CD83 and fluorescein isothiocyanate anti-CD86 (BD Biosciences) for 30 min at 4°C. Cells were washed in wash buffer before analyses of the percentages and MFIs of cells expressing activated DC markers.

### T-cell proliferation assays

Allogeneic T cells were washed in warm PBS and cultured with 1 *μ*M carboxyfluorescein succinimidyl ester (CFSE; Invitrogen, Paisley, UK) in basal culture medium for 10 min at 37°C. Dye was then quenched by washing thrice in ice-cold medium. DCs that had been pre-stimulated with tumour-derived supernatant were then admixed with CFSE-loaded T cells at a ratio of 1 : 10 (affector DCs:effector T cells), and cultured in a humidified atmosphere of 5% CO_2_ at 37°C for 4 days. The magnitudes of CFSE-positivity of the T cells were then assessed by flow cytometry.

## Results

### Exposure to chemotherapy

Three chemotherapeutic drugs commonly used in the treatment of solid tumours were investigated in this study and selected on the basis of their reported associations with immune modulation. There were dose-dependant reductions in cell viabilities in cultures with CPM, OXP and GEM (Table 1). Approximate IC25 concentrations were extrapolated on Emax curves and used in the *in vitro* experiments. In the murine study, drugs were used at clinically relevant concentrations as described in the literature.

### HLA1 expression is increased by chemotherapy

**Figure 1:**
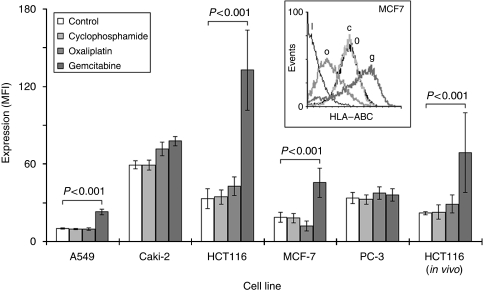
Chemotherapy increases human leucocyte antigen class 1 (HLA1) expression in tumour cells. Tumour cell lines were cultured with equi-active ∼IC25 concentrations of each of the drugs for 3 days before the assessment of HLA1 (HLA–ABC) expression by flow cytometry. Representative histograms of expressions in MCF7 are shown (inset), where I = isotype control, 0 = control, o = oxaliplatin, c = cyclophosphamide and g = gemcitabine. Each data column is a representative of the mean and s.d. of at least three separate experiments. *In vivo* HCT116 data generated from the mean and s.e.m. of five mice.

Human leucocyte antigen class 1 expression in cells was assessed by flow cytometry using a proprietary antibody directed against HLA–ABC and presented as MFI relative to the isotype control. They ranged from 10±0.67 in A549 to 59±3.1 in Caki2, and divided into those with low expression (A549 and MCF7) and those with high (Caki2, HCT116 and PC3) (Figure 1). There was little effect on HLA1 expression of culturing cells with ∼IC25 concentrations of CPM or OXP. However, culturing with GEM caused significant increases within HCT116, A549 and MCF7 cells (MFI cf. untreated controls: 132±30 *vs* 33±7.8; 0.23±2.3 *vs* 10±0.67; and 45±11 *vs* 18±3.7, respectively; *P*<0.01) (Figure 1).

Our *in vivo* model showed HLA1 expression in HCT116 growing subcutaneously in mice was also increased after treatment with GEM (*P*<0.001 cf. untreated controls), but remained unchanged when treated with CPM or OXP (Figure 1). This was seen just after 3 days of intra-peritoneal administration of drugs. No significant changes to tumour masses were observed after this short time-course (data not shown).

### HLA1 expression is associated with cytotoxic T-cell function

**Figure 2:**
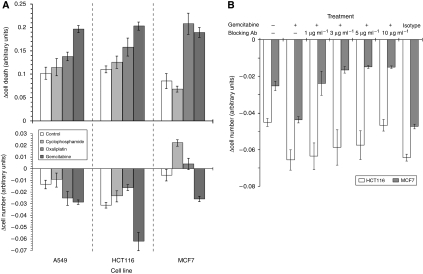
Human leucocyte antigen class 1 (HLA1) expression is associated with cytotoxic T-cell function. A549, HCT116 and MCF7 cell lines were cultured with equi-active ∼IC25 concentrations of each of the drugs for 3 days. Drugs were then removed and the cells were washed before being admixed with cytotoxic T cells at a ratio of 20 : 1. (**A**) Cultures were maintained for a further 24 h before the assessment of cell death/viability by the lactate dehydrogenase (LDH) and methylthiazoletetrazolium (MTT) assays. There were significant increases in cytoxicity (LDH: upper panel) and concomitant decreases in cell number (MTT: lower panel) in those cultures where gemcitabine was used. (**B**) Blockade of HLA1 in HCT116 and MCF7 cells by using antibodies negated the cell death associated with increased HLA1 expression. There was little change to cytotoxicity in cells treated with the isotype control antibody, which was used at 10 *μ*g ml^−1^. Each data column is a representative of the mean and s.d. of at least three separate experiments.

We investigated the effect of HLA1 expression on the functional ability of cytotoxic T cells to induce cellular death by subjecting those tumour cells with drug-increased HLA1 to the cytotoxic effects of a modified mixed lymphocyte reaction. Results indicated reductions in cell number and a concomitant increase in cell death after exposure to supernatant derived from OXP or GEM as assessed by the MTT and LDH assays, respectively (Figure 2A). These effects were most clear with cells cultured in GEM-derived supernatant, in which the changes in HLA1 expression were most pronounced. Blockade of HLA1 with an antibody (clone W6/32) in cells showing the greatest cytotoxic effect (HCT116 and MCF7) reduced the extent of death. This was achieved at a lower concentration of 1 μg ml^−1^ in MCF7 cells and at 10 μg ml^−1^ in HCT116 (Figure 2B), and coincided with the extent of increased HLA1 expression.

### Supernatants from tumours treated with chemotherapy stimulate DCs

Initial flow cytometric analyses (data not shown) of the effects of supernatants derived from tumour cells treated with chemotherapy on peripheral blood mononuclear cells showed no changes in MFI or percentage of cells in the natural killer (NK), T-cell and regulatory T-cell (T-reg) subsets. However, there were changes in the monocyte population, and so we explored more carefully, the effects of the supernatants on professional APCs from this fraction. Our plastic adherence method of DC-generation resulted in high yields (∼80% of total event population – based on FSC and SSC patterns) and the purities of the DCs (CD11c+, HLA–DR+ and CD14^low^) were >95%. The presence of undifferentiated monocytes was low with an average CD11c+ and CD14^high^ signal of 1.5%.

**Figure 3:**
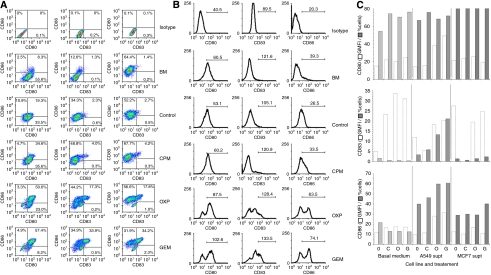
Supernatants from tumours treated with chemotherapy stimulate dendritic cells (DCs). DC-derived monocytes were cultured with exhausted culture media from A549 and MCF7 treated cells. The geometric mean fluorescence intensities (GMFIs) and percentages (percentage of cells) of expressions of the maturation markers CD80, CD83 and CD86 were assessed after 24 h by flow cytometry. Representative density plots with %cells distribution within each quadrant (**A**) and histograms highlighting the GMFI within the defined range (**B**) are shown for DCs exposed to A549-derived media. These media were from cultures treated with cyclophosphamide (CPM), oxaliplatin (OXP) or gemcitabine (GEM). Tumour-free basal medium (BM) and media from untreated tumour cells (control) were also included. (**C**) There were generally increases in the markers (either in terms of GMFI and/or percentage of cells) upon exposure to supernatants from cyclophosphamide (C), oxaliplatin (O) or gemcitabine (G) compared with untreated tumours (0), which was most clear in the A549 cohort of samples. Each column is a representative of the mean of at least three separate experiments and s.d. has been omitted for clarity.

Culturing the DCs with chemotherapy alone resulted in no significant changes to CD80, CD83 and CD86 expressions as defined by both percentage of positive cells (percentage of cells) and MFI (Figure 3C). Although there were significant increases in these differentiation markers on culturing DCs with supernatant derived from A549 and MCF7 tumours, there were further increases in expressions when the supernatant were from tumour cells treated with chemotherapy (Figure 3A-C).

### Supernatant-stimulated DCs enhance the proliferation of allogeneic T cells

**Figure 4:**
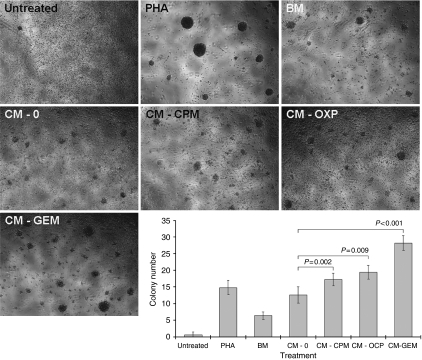
Supernatant-stimulated dendritic cells (DCs) increase the number of T-cell colonies. DCs matured with supernatant (CM) derived from A549 treated with cyclophosphamide (CPM), oxaliplatin (OXP) or gemcitabine (GEM) were admixed with allogeneic T cells and the number of colonies present enumerated on day 4. There were significantly larger numbers of colonies in wells containing DCs pre-exposed to chemotherapy-treated tumour. Typical pictures of colonies seen in each condition are shown, and each data column. Each data point is a representative of the mean and s.d. of five separate experiments.

**Figure 5:**
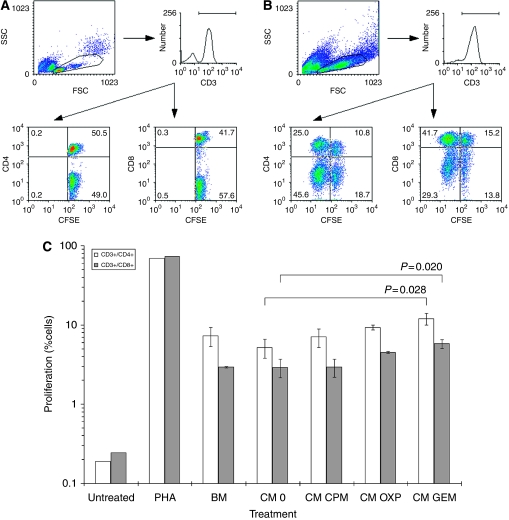
Supernatant-stimulated DCs increase the proliferation of T cells *in vitro*. DCs matured with supernatant (CM) derived from A549 treated with cyclophosphamide (CPM), oxaliplatin (OXP) or gemcitabine (GEM) were admixed with allogeneic T cells loaded with carboxyfluorescein succinimidyl ester (CFSE). (**A**, **B**) Proliferation as indicated by a downward shift of CFSE mean fluorescence intensity was increased after culture with the positive control of PHA. Both CD4+ and CD8+ subsets were assessed. (**C**) There was a trend of increased percentage of proliferating T cells cultured with DCs stimulated with CM, which was significant (*P*<0.03) in the samples involving GEM. Each data column is a representative of the mean and s.d. of three separate experiments.

Carboxyfluorescein succinimidyl ester-loaded T cells were admixed with DCs stimulated with tumour-derived supernatant and the effect on proliferation was assessed by enumerating T-cell colony numbers and flow cytometric analyses of CFSE-signals in T cells. Greatest changes in DC-markers were seen in A549 cells cultured with chemotherapy and so the ability of these DCs to stimulate T-cell proliferation was studied. Initial simple morphological examination of colonies showed significant increases in their number in cultures with DC pre-treated with supernatants derived from tumours (Figure 4). Results were recapitulated in the CFSE-loaded T-cell experiments, which showed increases in the percentage of proliferating CD3+/CD4+ and CD3+/CD8+ T cells cultured with DCs derived from exposures to basal medium (7.3±1.9% and 2.9±0.059%, respectively), which were not significantly different to proliferation seen in T cells admixed with DCs exposed to supernatant derived from untreated A549 tumour (Figure 5A, B). T-cell proliferations were further increased when the DCs were pre-exposed to chemotherapy, which was significant when using supernatant from tumours cultured with GEM (Figure 5C).

## Discussion

This study was undertaken as part of our larger remit to investigate whether immunotherapies could enhance the activities of other modalities and, thus, improve the outcome and quality of life in cancer patients. In this study, we specifically investigated the immunopotentiating effects of CPM, OXP and GEM on a panel of cancer cell lines. By using *in vitro* models, we could assess the effects of these traditional chemotherapeutic agents on immune cells and tumour cells in isolation. In summary, our results showed that these cytotoxic drugs had the supplementary effect of potentially enhancing adaptive immune responses through a dual effect of increasing DC function that augmented a T-cell response and partial restoration of immune-visibility of tumour cells to T cells by an upregulation of HLA1 expression.

Enhancing the body's natural ability to trigger the immune system to kill cancer cells underlies the principle of biological therapies. These can take the form of interferons (IFNs) and growth factors, as well as a DC-vaccine approach (Dalgleish, 2004; Copier and Dalgleish, 2006), which are administered as an attempt to stimulate or restore the ability of the immune system to fight disease (Weiner *et al*, 2009). Recently, chemotherapeutic agents have been added to the list of biological response modifiers. These drugs are not tumour specific and affect other rapidly proliferating cells and so consequently, bone marrow-derived haematopoeitic progenitor cells will also be affected, which reduces blood cell function. Therefore, chemotherapy should be immunosuppressive; however, it can have the opposite effect and actually enhance immune function (Chaudhuri *et al*, 2009).

The underlying mechanism of immunopotentiation for most chemotherapeutic drugs is unknown but paradoxically, may be a consequence of their cytotoxic and immunosuppressive effects. For example, the cytotoxic alkylating agent CPM can also selectively inhibit T-regs (Meyer *et al*, 2009). This effect coupled with the intrinsic response of immune stem cells to rapidly mobilise and replace lymphocytes lost to CPM, can induce a quasi-hypercytokinemic event that ‘boosts’ the pool of functional T cells (Brode and Cooke, 2008). In addition, CPM may potentiate these T cells by supporting the production of T-cell-related growth factors such as type I IFNs (Schiavoni *et al*, 2000). These immunostimulatory events have been known since the mid-1980s, and originate from a direct effect of the drugs on immune cells (Berd *et al*, 1984; Livingston *et al*, 1987). However, little is known about the indirect effects of chemotherapy on immunity. Indeed, their cytotoxic effect towards tumour cells would generate a rich source of antigens and cytokines, which could benefit and support immune responses. For this reason, we explored the concept that the exhausted culture media from cells treated with chemotherapy may be immunologically active.

Preliminary experiments showed that supernatants from tumours treated with any of the drugs had no direct effect on the numbers and/or activation states of NKs (CD3−, CD69+, CD56+ and CD16+), T cells (CD3+, CD25+ and CD69+) and T-regs (CD4+, CD25+ and Foxp3+). However, significant changes were seen in the professional APCs (CD3−, CD80+, CD83+ and CD86+) population (data not shown). Consequently, we explored more carefully, the effects of these supernatants on monocyte-derived DCs. DCs have an important role in the development of adaptive immune responses to antigens by influencing the differentiation of naive T cells into effector T cells (Steinman, 1991). Treatment of these DCs with supernatants derived from MCF7-treated tumours had no significant effect of DC markers. However, treatment with those derived from A549 resulted in a greater extent of maturation and increased expression of co-stimulatory markers (MFIs and/or percentage of cells), which indicated immune activation (Dilioglou *et al*, 2003). Each of the drugs was used at equi-toxic concentrations and the effect was drug-specific, with GEM-derived supernatant being most DC-maturing and CPM the least.

After antigen uptake and processing, mature DCs migrate to sentinel lymph nodes where they can interact with naive T cells. Therefore, we next tested the functional ability of these mature DCs to engage allogeneic T cells by using a modified mixed lymphocyte reaction, in which DCs pre-exposed to supernatant were admixed with allogeneic CFSE-loaded T cells (Lyons, 2000). The A549 tumour cell line was only used in these experiments as they exhibited greatest change in CD80/83/86 expression, and so would be most likely to activate T cells. Results showed increased proliferation of CD3+/CD4+ and CD3+/CD8+ cells in cultures wherein the supernatants were DC-maturing, which were significant where maturation was greatest (GEM-derived supernatant). Increased proliferation was also associated with increased production of IFN-*γ* (data not shown).

Dendritic cells are specialised APCs that are able to process and present antigens to T cells, which enables the immune system to mount an antigen-specific immune response against tumour cells. They are commonly used as vaccines (Mocellin *et al*, 2004). DC-vaccine therapy involves generating immature DCs *in vitro* by culturing peripheral blood monocytes with GMCSF and IL4, before exposing them to cancer-specific antigens and re-administration into the patient. Maturation is essential for eliciting an immune response (de Vries *et al*, 2003) and is commonly induced by culturing in a cocktail of tumour necrosis factor-*α*, IL-1*β*, IL6 and prostaglandin-E2 (Jonuleit *et al*, 1997). These cytokines are used to prepare vaccines for cancer patients; however, our results suggest that maturation may be achieved by exposing immature DCs to supernatants derived from tumours without the need for other growth factors. Also, these supernatants are rich sources of tumour-derived antigens that can support a cytotoxic T-cell response. Similarly, the strong possibility that tumours cells challenged with chemotherapy are producing cytokines that potentiate DC function is interesting, and forms the basis of an ongoing parallel study. Although our results stem from allogeneic experiments, the idea that supernatants derived specifically from tumours treated with some forms of chemotherapy can enhance adaptive immune responses is attractive. These data support the idea of combinations between immune-based and conventional cancer therapies, and so we are currently investigating these effects in an autologous system.

A decrease in the expression and/or function of HLA1 has often been found in tumour cells, which may provide a route through which adaptive T-cell responses can escape (Cabrera *et al*, 2003; Aptsiauri *et al*, 2007). The deficiency can be caused by loss of transcription and translation elements (Garrido *et al*, 1993) or epigenetic modifications that silence regulatory genes (Tomasi *et al*, 2006). Losses can also result from dysregulations of cellular signalling pathways, which are commonly altered in cancer cells. Parenthetically, these pro-oncogenic events can, in addition to promoting tumour growth and survival, reduce immune visibility (Bianco *et al*, 2006). HLA1 expression was assessed in our small group of tumour cell lines and the basal levels varied from relatively high (Caki2) to low (A549). As cytotoxic T cells are restricted by HLA1 and kill tumour cells only in the presence of HLA1, tumours lacking this antigen may be undetected by immune cells. Consequently, enhancing HLA1 expression could restore immune visibility in tumour cells. This has been shown possible by irradiation (Klein *et al*, 1994), hence we next assessed the effects of chemotherapy on HLA1 expression on tumours. Results showed that treatment with ∼IC25 concentrations of GEM could increase the expression of HLA1 in tumour cells; some of which exhibited low basal levels. HLA1 upregulation was recapitulated *in vivo* in GEM-treated HCT116 tumour cells grown subcutaneously in nude mice.

The cytotoxic effect of T cells on tumour lines with increased HLA1 expression was assessed by a modified mixed lymphocyte reaction, wherein allogeneic CD3+ sorted cells were admixed with tumours. The magnitude of cell killing was established by measuring LDH release from cells and by assessing cell numbers by the MTT assay. Results showed there was increased cytotoxic T-cell killing of those tumour cells with enhanced HLA1 expression, which was HLA1 specific, as cytotoxicity was reduced by using a blockade antibody (Fayen *et al*, 1998). Furthermore, antagonism was only effective at higher antibody concentrations in HCT116; a cell line in which HLA1 expression was increased the most. Technically, there was a general agreement between the assays, with increases in cytotoxicity associated with decreased cell numbers. There were some differences, for instance the increased LDH release in OXP treated MCF7 cells were not recapitulated by a decrease in MTT signal. The discrepancy between, and limitations in, the assays have been noted previously (Lobner, 2000; Fotakis and Timbrell, 2006; Liu and Dalgleish, 2009) and could be because of the washing steps that are part of the MTT assay, whereas the LDH assay uses the culture medium directly from the cells. Nevertheless, the possibility of increasing HLA1 in tumours that have low expression as a way of enhancing immune responses is appealing and is highlighted in this study.

Restoration of HLA1 expression can also have the opposite effect of rendering tumour cells refractory to NK cell mediated lysis. Specifically, the expression of a subset of HLA1 molecule can disrupt NK activity, thereby allowing tumour cells to escape innate immune responses (Storkus *et al*, 1991). Consequently, a reduction in its expression may be a more favourable event. Our results also showed that in addition to increases in HLA1 expression, some drugs used in the correct tumour type could reduce HLA1 expression (i.e. OXP in MCF7; Figure 1). This presents the fascinating possibility of mixing and matching chemotherapies to modify HLA1 expression in a way to exploit both the adaptive and innate immune responses (Rees and Mian, 1999). This is currently being investigated.

In summary, early studies have suggested that the induction of an effective immune response could lead to the elimination of residual tumour. Subsequent attempts to enhance the immune response have essentially been on vaccine basis, trying to induce a specific response against the tumour. Numerous vaccine approaches have claimed to provide significant clinical benefits, but very few of these have survived a randomised trial. Much is known about barriers to effective vaccine therapy and it is generally assumed that ‘vaccine with something else’ is best. With our improved understanding that a number of chemotherapeutic agents have, in addition to their conventional anti-cancer effects, an element of immune stimulation means that treatment strategies can be developed to exploit the duality of these drugs. Using chemotherapy as an adjuvant to immunotherapy could work in a number of ways. Tumour burden would be reduced through its direct cytotoxic effect, which would simultaneously generate a source of cancer-derived antigens in the form of cellular debris. Furthermore, it could restore immunovisibility by increasing HLA1 and simultaneously enhance the response of the immune system to immunotherapies.

## Figures and Tables

**Table 1:** IC50 values. Cell lines were cultured with each of the drugs for three days before the assessment of the concentration required to reduce cell numbers by 50% by the methylthiazoletetrazolium (MTT) assay

**[*μ*M]**	**Cyclophosphamide**	**Oxaliplatin**	**Gemcitabine**
A549	>1000 (100)	12±8.9 (1)	13±2.8 (1)
Caki2	>1000 (300)	33±14 (3)	13±8.6 (1)
HCT116	>1000 (100)	27±23 (1)	7.2±4.2 (0.6)
MCF7	>1000 (100)	7.6±7.2 (0.6)	2.3±0.45 (0.3)
PC3	>1000 (300)	16±12 (3)	4.9±2.4 (0.6)

Values within the parentheses are ∼IC25 concentrations as determined by Emax-fitted curves. Values are in *μ*M and represent the mean and s.d. of at least four separate experiments.
